# Establishment of a post-disaster healthcare information booklet for the Turkey–Syrian earthquake, based on past disasters

**DOI:** 10.1038/s41598-024-52121-4

**Published:** 2024-01-18

**Authors:** Junko Okuyama, Shuji Seto, Yu Fukuda, Yoshimi Suzukamo, Tatsuma Okazaki, Yoshihito Furusawa, Shin-Ichi Izumi, Kiyoshi Ito, Fumihiko Imamura

**Affiliations:** 1 Core Research Cluster of Disaster Science, Tohoku University, Miyagi, Japan; 2 Department of Rehabilitation, Tohoku University Hospital, 1-1 Seiryo-machi, Aoba-ku, Sendai, Miyagi 980-8574 Japan; 3 Office for Establishment of New Faculty, Akita University, 1-1 Tegatagakuen-machi, Akita-shi, Akita 010-8502 Japan; 4 Tsunami Engineering Lab, International Research Institute of Disaster Science (IRIDeS), Tohoku University, 468-1 Aoba, Aramaki-aza, Aoba-ku, Sendai, Miyagi 980-8572, Japan; 5 Notre Dame Seishin University, 2-16-9 Ifuku-cho, Kita-ku, Okayama City, Okayama 700-8516, Japan; 6 Department of Physical Medicine and Rehabilitation, Tohoku University Graduate School of Medicine, 1-1 Seiryo-machi, Aoba-ku, Sendai, Miyagi 980-8574 Japan; 7 Disaster Obstetrics and Gynecology Lab, International Research Institute of Disaster Research (IRIDeS), 468-1 Aoba, Aramaki-Aza, Aoba-ku, Sendai, Miyagi 980-8572 Japan

**Keywords:** Psychology, Natural hazards, Health care

## Abstract

The scientific evidence based on experiences with past disasters points to the possibility of the occurrence of future mental health issues among those who were affected by the recent Turkey–Syria earthquake. However, post-disaster care information on factors that could give rise to mental health issues among those affected have yet to be provided. In March 2011, Tohoku University compiled and published a booklet with post-disaster healthcare information based on the experiences with the Great East Japan Earthquake. This study aimed to promote the introduction and use of this booklet for post-disaster care in Turkey and Syria by presenting the results of a satisfaction survey conducted with relevant Japanese organizations about the booklet. A total of 505 Japanese organizations participated in the satisfaction survey of, and evaluated, the booklet. The results indicated the need to consider the ease of understanding for the general public when providing information on post-disaster care through booklets. We hope that this study leads to the appropriate provision of easy-to-understand, post-disaster healthcare information to the victims of the Turkey–Syria earthquake and future disasters.

## Introduction

A major earthquake that struck southeastern Turkey and northern Syria on February 6, 2023, has created a humanitarian crisis^1^. The earthquakes struck ten Turkey provinces that together correspond to roughly the size of Austria, affected approximately 15 million people, damaged more than 200,000 buildings, and resulted in the collapse of about 20,000 buildings^2–4^. Moreover, the number of deaths and scale of the damage may continue to grow, especially in northwestern Syria, where search and rescue operations have been hampered owing to border restrictions. However, without careful mitigation, the people of these two countries will suffer long-term health consequences^5^. Worldwide, some of the regions that frequently experience earthquakes are China, Indonesia, Iran, and Japan. Earthquakes cannot be prevented from occurring, but their disasters can be mitigated by proper preparations. Despite the recurrence of earthquakes worldwide and the acknowledged importance of preparedness, the effective implementation of lessons learned from recent major earthquakes remains a challenge^6^.

Natural and human-made disasters are known to have a considerable impact on the mental health of those who experience them. Accordingly, several studies have been conducted on the prevalence of psychopathology in disaster-affected populations, delivering relevant evidence to be used by service planners, regarding the nature and magnitude of services required in the event of different disasters^7,8^. For example, previous studies on disasters in Asian regions since the 2004 Indian Ocean earthquake and tsunami events demonstrated the effectiveness of cognitive–behavioral therapy for treating post-traumatic stress disorder in Asian survivors; however, this type of therapy must be adapted to cultural and regional sensitivities^9,10^. In another report, researchers explored the recommended actions for dealing with the Turkey–Syria Earthquake based on past experiences from the Great East Japan Earthquake, advising the need to secure direct support routes and dispatch of medical personnel to the affected areas^11^. While these recommendations outline the actions to be taken during the aftermath of a disaster, the projections about the burden of diseases related to a disaster event may change across the phases of the event and disaster management, and these projections are essential for guiding medical responses^12^.

**Figure 1:**
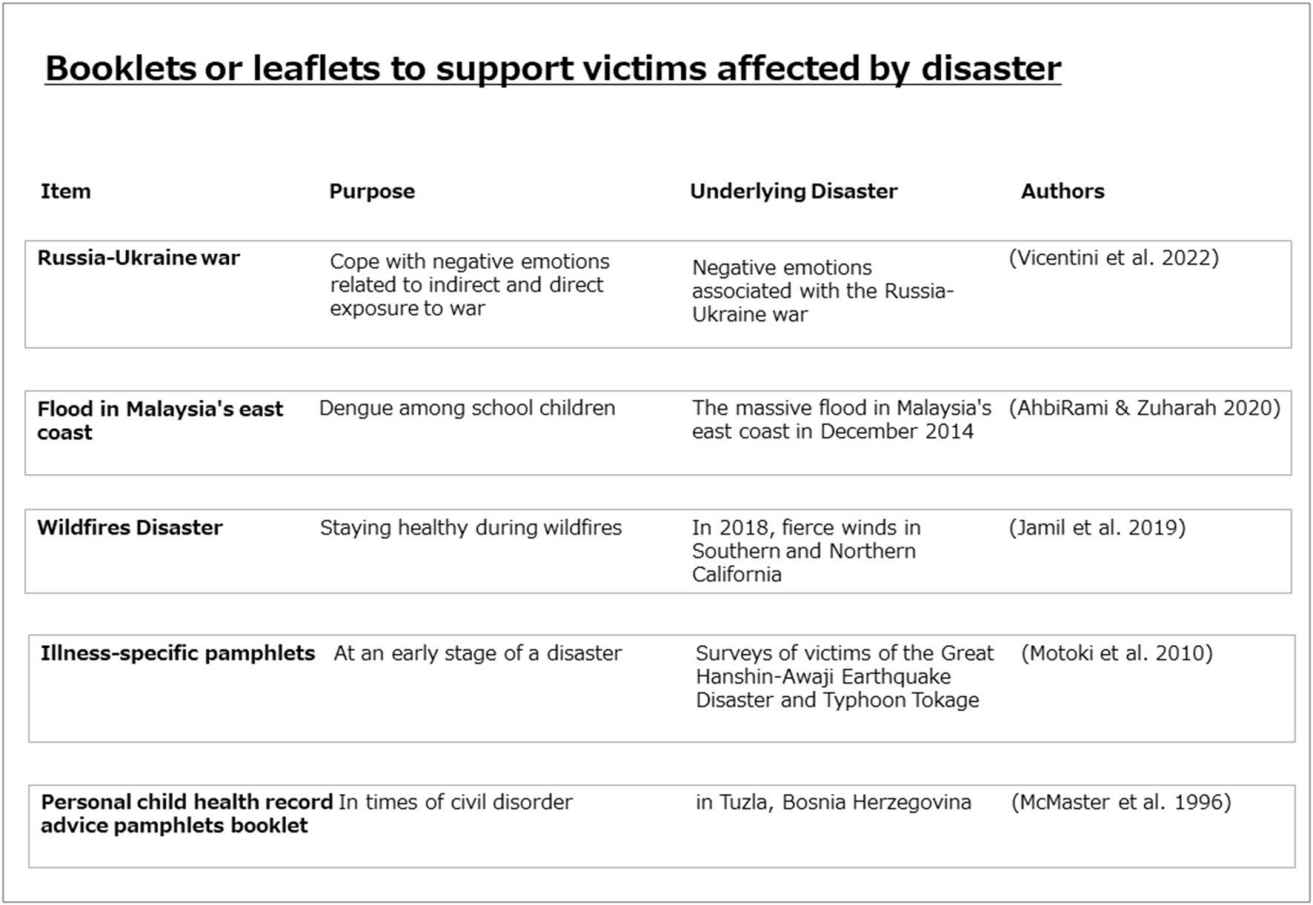
Booklets or leaflets to support victims affected by disaster. A search on PubMed (March 26, 2023) was conducted to identify past studies on booklets or leaflets used to contribute to post-disaster care. From right to left, the figure shows the name of the booklet/leaflet, its purpose, the disaster on which it was based, and the authorship information of the relevant article.

Research emphasizes that medical and social service professionals should work with other key stakeholders to take the necessary actions to alleviate health problems in and help communities affected by disasters^13^. Materials such as booklets or leaflets designed to help survivors may be used in such alleviation efforts. For instance, an information booklet was used during the 2014 civil unrest in Tuzla, Bosnia Herzegovina^14^. Jamil et al. also created a booklet on “Guidance for protecting lungs from smoke from the 2018 California Wildfires”^15^. A booklet on dengue fever was distributed to prevent the spread of dengue after an outbreak caused by severe flooding on the east coast of Malaysia in December 2014^16^. Meanwhile, Vicentini et al. developed and evaluated psychoeducational materials to help children and adolescents cope with the negative emotions associated with indirect and direct exposure to war^17^. Figure 1 shows examples of these materials obtained from a PubMed search conducted on March 26, 2023.

It is expected that university and research institute experts will use the scientific findings on disasters, post-disaster reconstruction, and engage in booklet production. However, the general public can also benefit from such booklets. Albeit, this requires the production of easy-to-understand booklets and the presentation of research findings in a manner that is acceptable to a broad audience. In Japan, local governments have been and continue to be the responsible bodies for leading post-earthquake reconstruction measures^18,19^, and this contextual reality makes considering the expertise of Japanese municipality governments an important effort when attempting to improve the contents and accessibility of booklets for the public.

**Figure 2:**
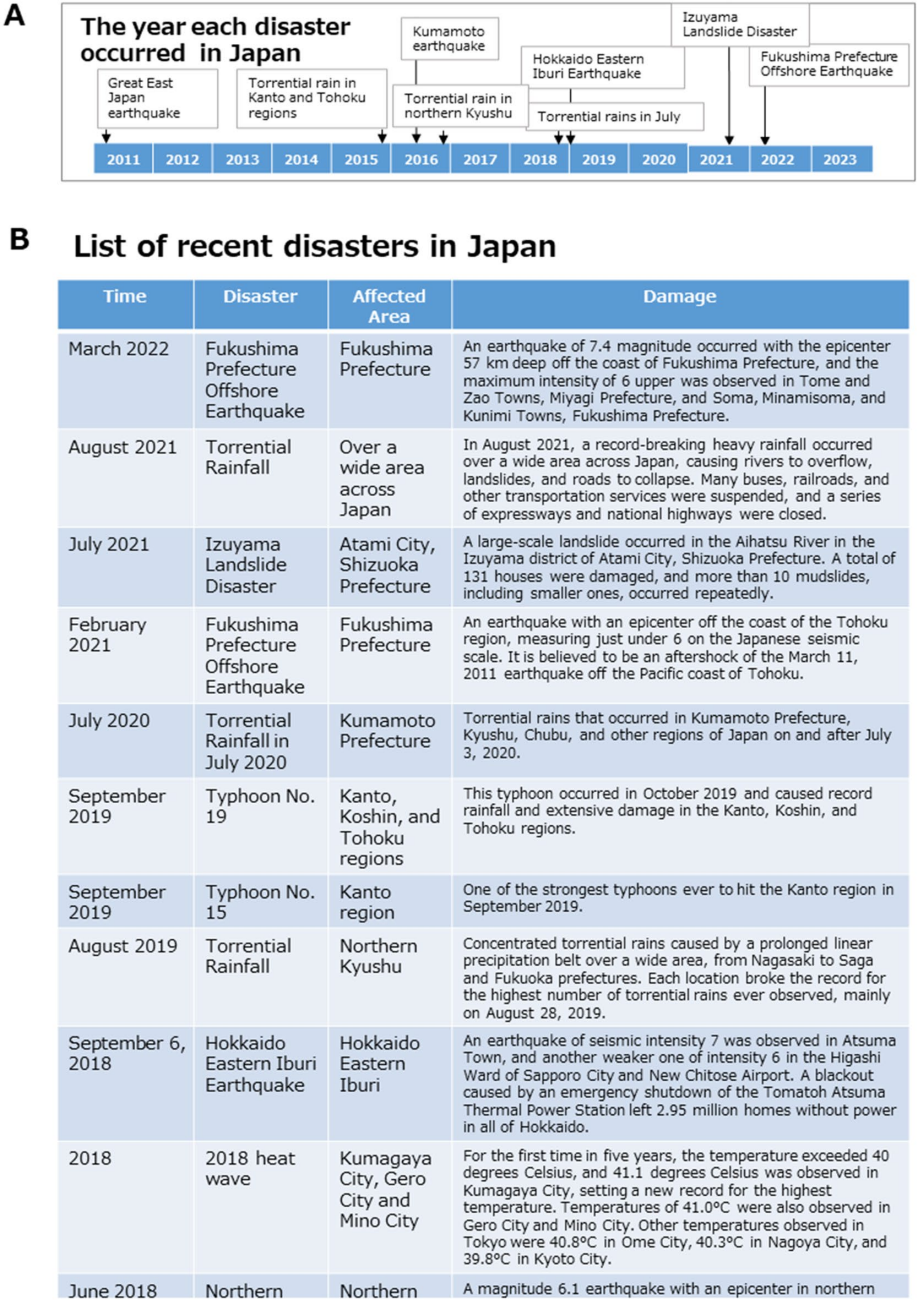
List of recent disasters in Japan. This figure shows the major natural disasters that have occurred in Japan since the Great East Japan Earthquake by year of occurrence. (**A)** Japan’s natural disasters by year. (**B)** Demographics of the disasters.

In Japan, natural disasters are a major threat, and weather disasters and earthquakes continued to occur in the country since the Great East Japan Earthquake on March 11, 2011 (Fig. 2A). It is also the case for Japan that climate change and its outcomes are amplifying the existing and creating new risks for both natural and human systems. The risk of weather-related disasters in Japan was unevenly distributed in few areas, with significant consequences for the people and society living there (Fig. 2B)^20^. Furthermore, post-disaster recovery measures tend to have a long-lasting impact on the lives of people living in affected areas. The distribution of literature on post-disaster living has been a common means of meeting the requirements for warning and informing the public about disaster and post-disaster preparedness. Such literature is often produced to meet mandatory requirements, such as of being understandable, informative, and encouraging compliance with desired behaviors; yet, information on the effectiveness of this literature is scarce. One thing we know based on past evidence is that the content, design, format, and presentation of information in such literature tends to be guided by ad hoc needs rather than by research evidence^21^.

In Japan, booklets have been prepared for disasters since the Great Hanshin-Awaji Earthquake of January 17, 1995. In fact, the booklet “Children Living in the Disaster Area: What Nursing Staff Can Do,” originally created after the Great Hanshin-Awaji Earthquake, was used to understand the situation of children affected by the 2004 Niigata-Chuetsu Earthquake^22^. Since then, based on experiences from the Great East Japan Earthquake of 2011^23^, disaster booklets have been produced in various parts of the nation. It is important to understand and explore the experiences from past disasters to reduce the impacts of future ones, and because the damage caused by a disaster can spread quickly, there is often the need to transmit the required information regarding disaster recovery for the population in a timely manner and at low costs. These characteristics of disasters and disaster management help transmit information in booklets, as these tools enable the provision of information for the population in a timely manner and in relatively cost-effective ways.

Therefore, booklets on disasters can be seen as links between what is known in academia about disaster recovery and the lives of people in affected areas. However, to date, no study has examined whether disaster-related booklets are suitable for the general public. Against this backdrop, this study aims to examine how academic knowledge is accepted by residents of disaster-affected areas through evaluations by local governments and other organizations of disaster booklets created by Tohoku University after the Great East Japan Earthquake. We specifically report on the results of an evaluation by local governments and organizations of a post-disaster care booklet and its potential use in populations affected by the Turkey–Syria earthquake. This evaluation was focused on assessing the kinds of booklets that are useful for promoting healthcare among people affected by the earthquake.

## Methodology

### Booklet evaluated

The booklet “Post-Disaster Health Care: ten issues to keep in mind to facilitate mental and physical recovery after a disaster” (hereafter, “Post-Disaster Health Care”), created by the Disaster and Health Unit of the Disaster Research Institute based on experiences of the Great East Japan Earthquake, was selected as the research subject. The following address allows you to download the English version of the “Post-Disaster Health Care” booklet: http://www.irides-pudh.med.tohoku.ac.jp/product/pdf/disaster_after_care/en/hisaigo_care-en_all.pdf. The purpose of creating the Japanese version of “Post-Disaster Health Care” and the Japanese version of “Post-Disaster Health Care” can be found on the website indicated in the Supplementary Information.

### Participants

**Figure 3:**
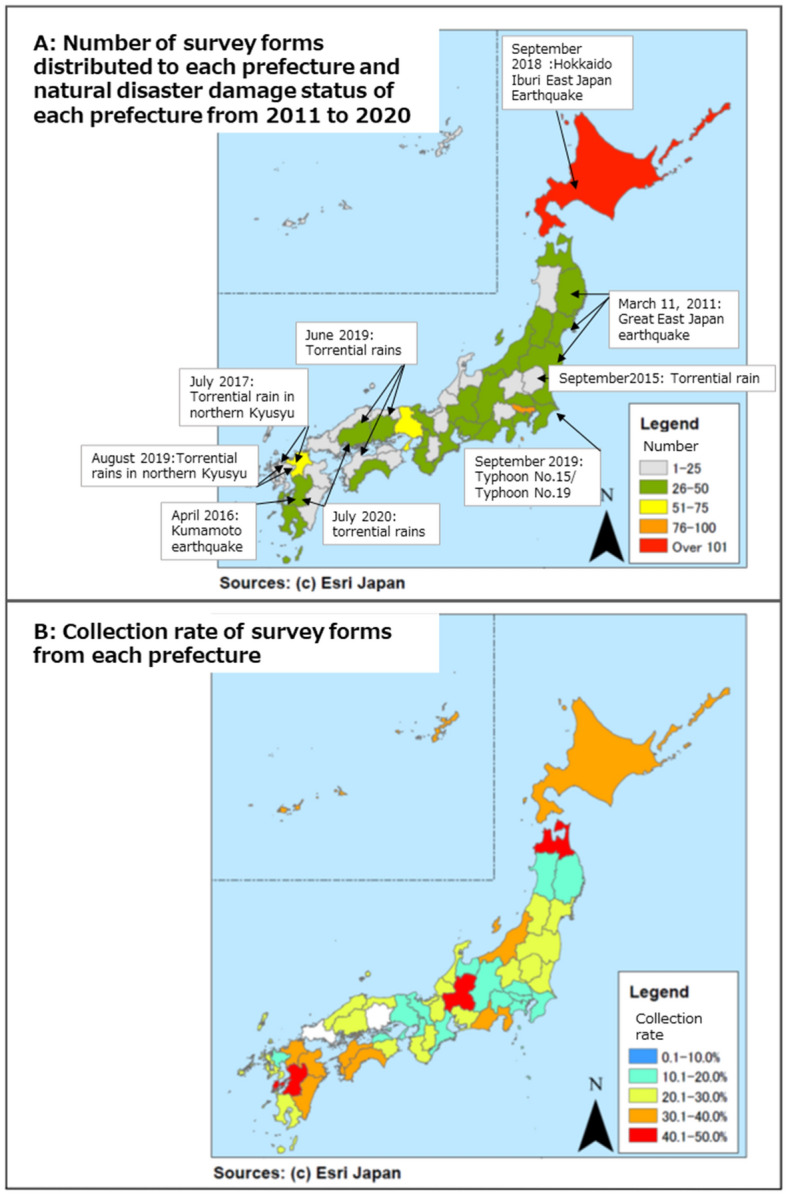
**(A)** Number of survey forms distributed to each prefecture and natural disaster damage status of each prefecture from 2011 to 2020. (**B)** Recovery rate of the questionnaire for each prefecture. Map background image source and license: Maps were created using ArcGIS Pro (ver. 3.1.2, https://www.esri.com/en-us/arcgis/products/arcgis-pro/overview) and the prefecture level boundaries of Japan were available in ArcGIS Hub. ArcGIS is the intellectual property of Esri and is used herein under license (Copyright Esri, All rights reserved). For more information about Esri software, please visit https://www.esri.com.

Around 1600 disaster-related locations in Japan were selected to receive disaster-related booklets. Fifty non-profit organizations and 28 mental healthcare centers were selected. A total of 175 places were selected, including 47 prefectures, 105 designated cities (20 government-designated cities, 58 core cities, and 27 special cities), and 23 special wards. Additional areas included 666 cities and 652 towns in the Sediment and Tsunami Disaster Law Warning Area. Figure 3A shows the number of questionnaires distributed per prefecture and the natural disasters that occurred in each prefecture.

### Materials

A questionnaire was designed to measure the ease of understanding of the “Post-Disaster Health Care.” To estimate whether the booklet information was effective, satisfaction was investigated for the whole booklet and each unit. The questionnaire was structured to assess the ease of understanding by asking respondents to rate their satisfaction with the booklet (five-point Likert-type scale ranging from 1, very satisfied to 5, very dissatisfied). Respondents were also asked to freely describe the reasons for their evaluations. Details on respondents’ sex, age, and workplace, among others, were also collected.

### Procedure

The questionnaires were mailed to 1,600 locations on January 30, 2020. They were delivered in envelopes that also contained the booklet “Post-Disaster Health Care,” an explanatory cover letter, and a return freepost envelope. Respondents were asked to send in an envelope if they were making a similar booklet to “Post-Disaster Health Care.” The respondents were asked to return the questionnaire by the end of February 2020.

### Ethical approval and consent to participate

All procedures contributing to this work were carried out in compliance with the ethical standards of the relevant national and institutional committees on human experimentation, and with the principles of Helsinki Declaration of 1975, as revised in 2008. This study was reviewed and approved by the Ethics Committee, International Research Institute for Disaster Science, Tohoku University; approval number: 2019-025, approval date: 22/JAN/2020, study title: “Satisfaction survey on post-disaster care (10 things to recover your mind and body).” All methods used in this study were carried out in accordance with relevant guidelines and regulations. Informed consent was obtained from all the participants included in the study. The purpose of the study was explained to each participant, and participation was voluntary; data collection was done anonymously.

## Results

### Response rate

In total, 505 questionnaires were returned (31.6% response rate), and 66.7% of the respondents were men and 22.2% were women (11.1% did not specify their sex). Regarding age group distribution, it was as follows: 20s (17.2%), 30s (28.1%), 40s (31.3%), 50s (16.6%), and over 60s (5.3%). The recovery rate for each prefecture is shown in Fig. 3B.

### Level of Understanding of the “post-disaster health care” booklet

**Figure 4:**
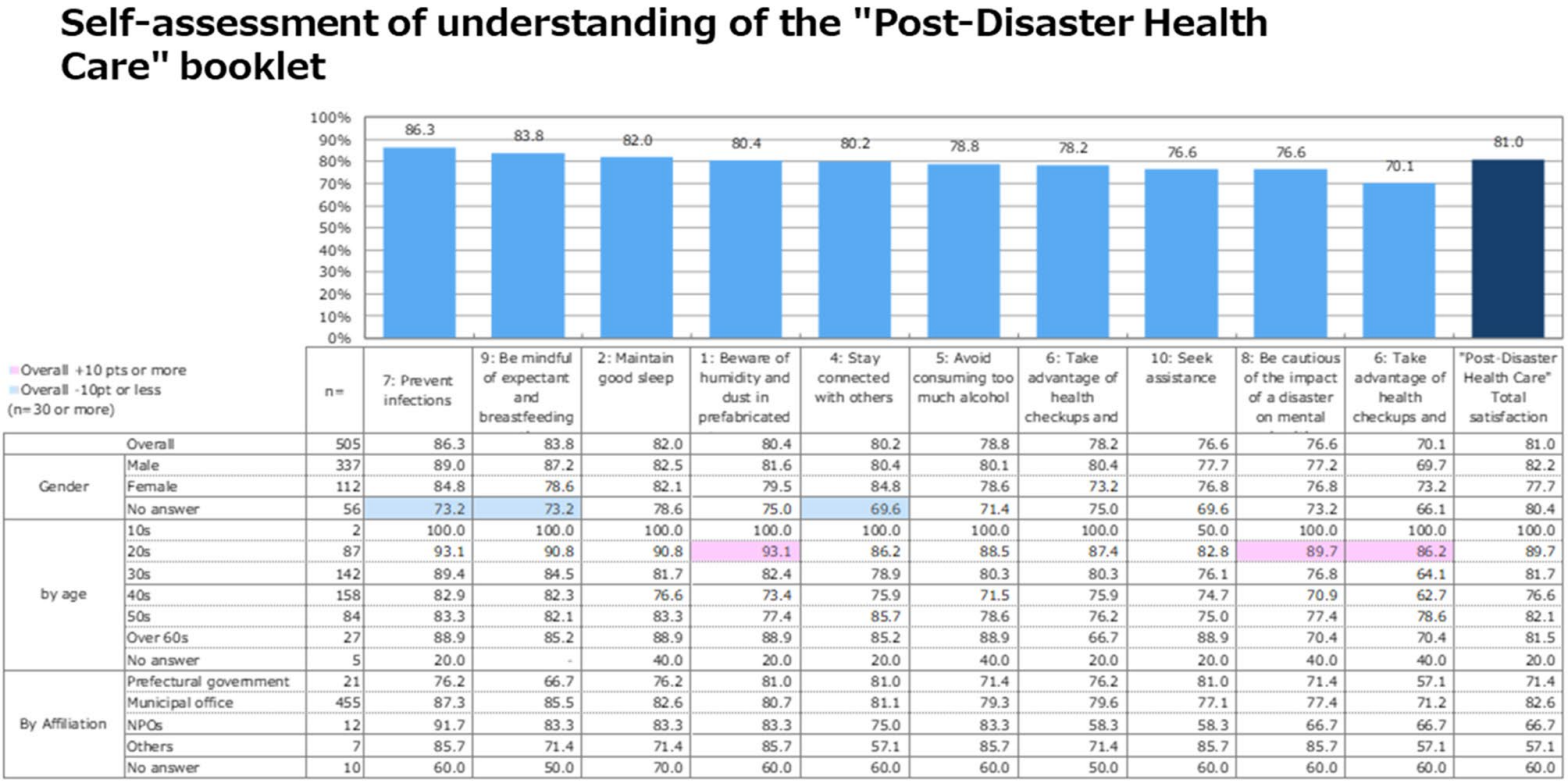
Self-assessment of understanding of the “Post-Disaster Health Care” booklet.

Figure 4 shows the level of understanding of the “Post-Disaster Health Care” booklet contents. Among the contents of the booklet, the highest level of understanding was for “prevent infectious diseases” (86.3%), which was followed by “be mindful of pregnant women and nursing mothers” (83.8%), “be careful of lack of sleep” (82.0%), and “be aware of humidity and dust in living spaces” (80.4%).

By age group, the percentage of respondents in their 20s who selected “Take advantage of health checkups and cancer screenings to take care of your health” was higher than the overall percentage. The overall satisfaction rate was 81.0%.

### Free responses analyzed by KH coder

**Figure 5:**
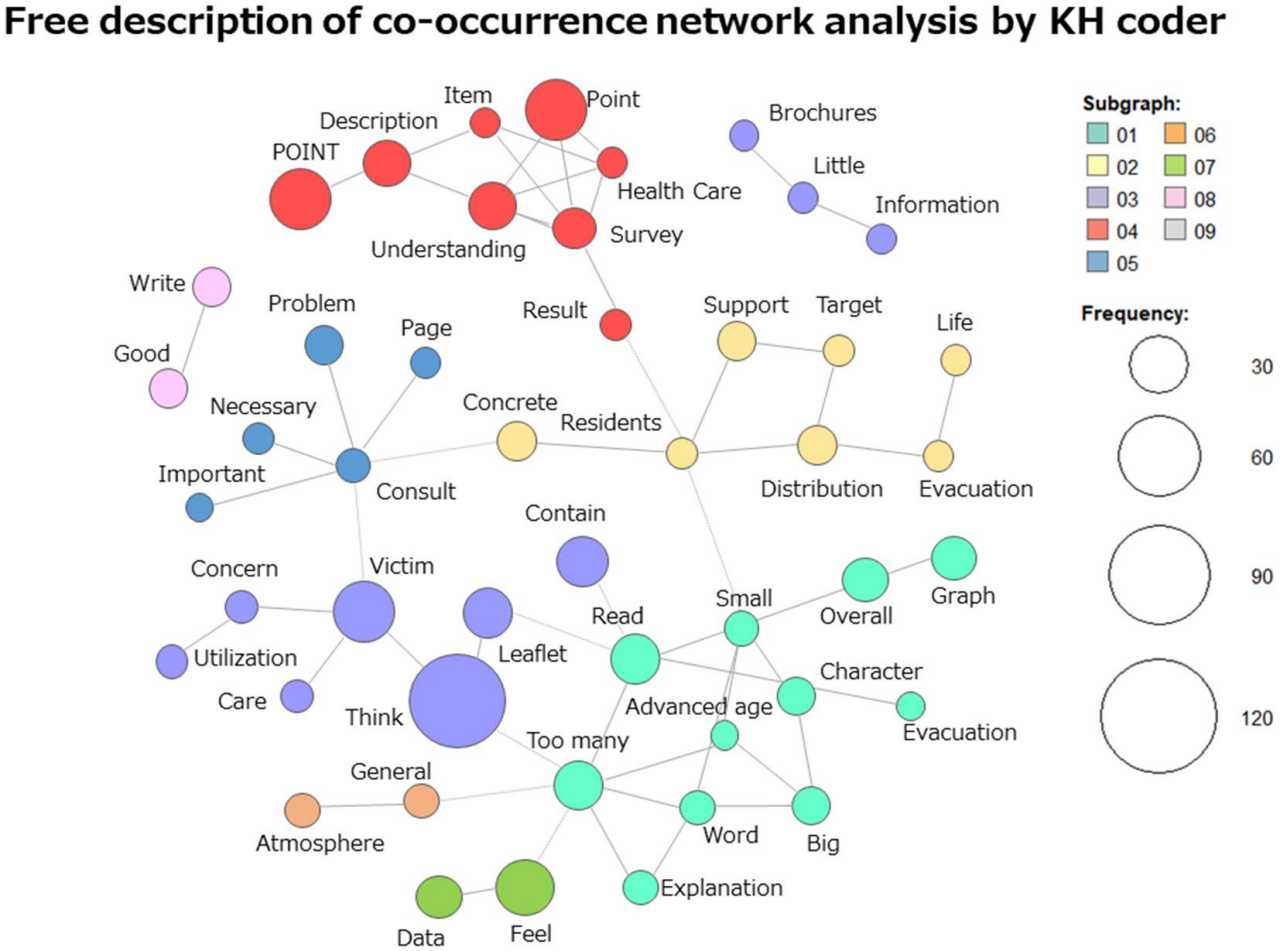
Free description of co-occurrence network analysis by KH Coder.

Figure 5 shows the co-occurrence network of the free responses extracted using KH coder. The responses were divided into nine clusters.

#### “Victim” “think” group

There were many comments, such as “I think there is a little too much text” and “I think it would be easier for people to accept easy-to-understand diagrams rather than graphs.” In summary, the general opinion was “I understand that the booklet is based on research results, but it may be difficult for the general public to read.”

#### “Point” group

This category includes questions such as “Who is the booklet intended for?” or “At what point after the disaster does it need to be read?” There was also a suggestion to devise a better and more consistent way to arrange the items, as they were not consistently arranged.

#### “Too many” “read” group

Since the booklet had many parts to be read, some asked for diagrams to depict the contents, and some said that the font was too small and difficult for older adults to read. There was an opinion that the graphs were difficult to understand and that what was described through graphs should instead be presented through illustrations.

#### “Distribution” “target” group

Many people said that if the information is intended for residents, they would rather have easy-to-understand content than the most accurate and specific information.

#### “Consult” group

Several respondents requested for consultation services to be specifically listed in the booklet.

#### “Brochures” group

Respondents wanted the booklet to be larger and have less information. There was a suggestion to increase the booklet size and its font size.

#### “Feel” “data” group

Many people commented that almost every page had data to support it and that it was persuasive.

#### “General” “atmosphere” group

Many people said that it would be difficult for the general public to accept the booklet.

#### “Good” “write” group

Many participants rated the content as good and well-written.

## Discussion

More than seven months have passed since the magnitude 7.8 earthquake struck southern Turkey and Syria on February 6, 2023^11^. Immediately after the earthquake, dozens of cases of crush syndrome were reported^24^, and risk factors for infectious disease outbreaks were concentrated in the affected areas in both countries. Tens of thousands of people were displaced as their homes either collapsed or were severely structurally damaged, making it impossible for them to return to their homes. A lack of access to safe drinking water, overcrowding, and poor sanitation resulting from the destruction of lifelines were the main risk factors of infectious disease outbreaks in the earthquake-affected areas of Eastern Anatolia; in addition, crises of various origins—such as the war conflict, the COVID-19 pandemic, and the cholera epidemic—coexisted and synergized with the disaster-related events^25^.

After the Turkey–Syrian earthquake, many researchers published insights on how to address the mental health needs of those affected^26–29^. Still, the effective prevention of mental health problems following disasters tends not to involve a major initial focus on mental health interventions right after the crisis. This is because affected populations tend to not be ready to receive mental healthcare in the immediate aftermath of a natural disaster^29^. Instead, the primary the focus should be on meeting the specific basic and physical needs of survivors, for whom there tends to be a lack of life-sustaining resources. Thus, comprehensive knowledge of post-disaster care is necessary when devising measures to alleviate pressure on health services, prevent mental health impacts on those affected, provide therapeutic benefits, build collective resilience to cope and, above all, recover from disasters. Our research has allowed us to present the evaluation of a booklet on post-disaster care developed based on the experiences of the Great East Japan Earthquake, which may be used by stakeholders for a more effective distribution of the limited medical resources after a disaster to victims in need of medical knowledge.

Few previous studies have examined the effectiveness of booklets on post-disaster care. One of the few related studies involved a telephone questionnaire to assess sources of health and safety information, recalled information, and behavioral responses among residents of the town of Fielding, New Zealand, following a major flood in February 2004^30^. The primary sources of information were radio (41%), brochures (23%), and newspapers (20%). This past evidence showcases that pamphlets/booklets are an important source of post-disaster health and safety information.

In the current survey, local government officials and others expressed a high level of satisfaction with the booklets prepared by Tohoku University experts (81.5%), albeit many expressed the opinion that the booklets may be difficult for the general public to understand. Although the graphs in the booklet showing the results of surveys conducted on the Great East Japan Earthquake are trustworthy, there were concerns that the public would not be able to understand their meaning and not know how to utilize the findings. Meanwhile, many preferred for the booklet to contain contact information for specific consultation services; if such information is indeed added, people would then be able to refer to a single source (i.e., the booklet) for information on whom to consult and the contact information, instead of having to search for contacts individually.

In Japan, local government officials are generally heavily involved in the collection of post-disaster public health information. Previous studies have considered the participation and knowledge of local government officials as important; for example, after the Great East Japan Earthquake, the knowledge of local government officials on radiation risks has been investigated^31^. Furthermore, while Japanese local governments have primary responsibility for disaster management, they often have limited capacity and experience in disaster management^32^. Therefore, it is a challenge for Japanese local government officials to learn from disasters that have occurred in other regions. This may explain why, in our sample, the “Post-Disaster Health Care” booklet was deemed useful by the officials for dealing with these challenges and facilitating the learning from other disasters. Most of the general public, in Japan and worldwide, obtain post-disaster information from TV, radio, and other media, and rarely obtain direct knowledge from experts^33^. These past findings once again underpin how the post-disaster booklet may be used and distributed by local government officials to further explain the situation to disaster victims.

Among the contents of the “Post-Disaster Health Care” evaluated in this study, the highest level of comprehension was for “prevent infectious diseases” (86.3%). This was clearly influenced by the COVID-19 pandemic, as the first case of coronavirus disease (COVID-19) had been detected in Japan on January 15, 2020, and the survey period for this study was shortly after this period (January 30 to February 28, 2020). In addition, the percentage of respondents in their 20s who responded that they took care of their health using health checkups and cancer screening was higher than the overall percentage. For the period after the Great East Japan Earthquake, only data on cervical cancer screening rates were presented in booklets when talking about cancer screening, which might have been a factor in the higher interest of people aged in their 20s. This may be because our survey was conducted during an ongoing campaign in Japan to encourage women aged 20–24 years to undergo uterine cancer screening^34^, as resistance to such screening has become an issue^35^.

The free text responses also revealed that it may be difficult for the public to accept the university- and scientific-based knowledge, which in turn was derived from past experiences of the Great East Japan Earthquake, relayed through the booklet. Specifically, participants remarked that the results should preferably be presented in concrete terms—rather than solely as graph data—to encourage the public to consider the issues. In addition, changing the design and font size may be beneficial for public acceptance of post-disaster care booklets.

The results suggest that more collaboration with the public is necessary to ensure the appropriate distribution of academic research knowledge to the public. Thus, it is beneficial for local governments, which serve as a bridge between universities and the public, to evaluate the contents of booklets. The findings of this study, which provide reference data based on the evaluations of governments and organizations of a post-disaster care booklet, may be useful for universities in making informed decisions about appropriate actions and initiatives for the benefit of local residents in disaster-affected areas in the future.

A limitation of this study is that the respondents were limited to Japanese local government officials, who in turn played and continue to play a central role in disaster management in Japan. Outside of Japan, local government officials do not always play a central role in disaster and post-disaster management, and they may not be appropriate respondents. While these limitations may have compromised finding accuracy, it is unlikely for this to have severely compromised the general finding patterns found.

### Supplementary Information

Supplementary Information.

## Data Availability

The “Post-Disaster Health Care” leaflet used in this document is available on the following website: http://www.irides-pudh.med.tohoku.ac.jp/product/pdf/disaster_after_care/en/hisaigo_care-en_all.pdf. Interview data is not available to the public due to the possibility of identifying individual participants. If you have any questions about the dataset or wish to obtain it under a data sharing agreement, please contact the first author.
