# Supplementary material for: Establishment of a post-disaster healthcare information booklet for the Turkey–Syrian earthquake, based on past disasters
Source: Sci Rep. 2024 Jan 18;14:1558. doi: 10.1038/s41598-024-52121-4 (PMC10796905; doi:10.1038/s41598-024-52121-4)

POINT 10

Seek assistance.

If you are experiencing severe and prolonged stress, or your mental and physical distress persists for a significantly long time, or you see no signs of improvement or have little support from those around you, please do not try to manage this alone and seek assistance by contacting a health care provider and/or local government office.

Even if the matter is small, talking it through with someone may help you find peace of mind in the path to recovery. Do not keep it to yourself, and find someone to talk to, including family, those you trust, and health care professionals. You can always recover from emotional and physical distress.

"Post-Disaster Health Care"

10 things that facilitate a mental and physical health recovery

Published on January 2019

指定国立大
災害科学 世界トップレベル研究拠点
Designated National University
The Core Research Cluster of Disaster Science

災害科学国際研究所
IRIDeS
International Research Institute of Disaster Science

Contributors: Hiroaki Tomita, Eiichi Kodama, Kiyoshi Ito, Shinichi Kuriyama, Shinichi Egawa, Koichi Chida, Masaharu Nakayama, Ken Osaka, Yoshio Hosoi, Halley Kaye-Kauderer

Disaster and Health Project Unit, Tohoku University

International Research Institute of Disaster Science (IRIDeS)

*Information in this booklet is valid as of January 2019.

*This booklet and its content is copyrighted by Tohoku University International Research Institute of Disaster Science (IRIDeS). Reproduction of part or all of the content without permission of the copyright holder is prohibited.

Post-Disaster Health Care

Ten issues to keep in mind to facilitate mental and physical recovery after a disaster

10

Introduction

After the 2011 Great East Japan Earthquake (GEJE), Tohoku University founded the International Research Institute of Disaster Science (IRIDeS). As a world-leading research center for natural disasters and disaster mitigation, IRIDeS sought to learn from and build upon past lessons in disaster management. IRIDeS is unique in that it integrates a division dedicated to research focused on disaster health science. The health division of IRIDeS has conducted numerous surveys to study the long-term impact of such a disaster with the hopes of extracting information beneficial for disaster response and recovery. In this leaflet, the following ten issues are advised to facilitate mental and physical health recovery from a disaster based on the long-term observations and surveys of the communities affected by the GEJE. While some of the recommendations may be specific to post-disaster settings in Japan (e.x. #1 or #6), many of them can be universally applied.

Points to Remember

1. Beware of humidity and dust in prefabricated temporary housing.
2. Maintain good sleep.
3. Maintain routine physical activities.
4. Stay connected with others.
5. Avoid consuming too much alcohol.
6. Take advantage of health checkups and cancer screenings.
7. Prevent infections.
8. Be cautious of the impact of a disaster on mental health.
9. Be mindful of expectant and breastfeeding mothers.
10. Seek assistance.

POINT 1

Beware of humidity and dust in prefabricated temporary housings.

Survey findings Increased prevalence of allergic diseases after the GEJE.

Recommendation Watch for indoor air quality in temporary housings.

In a survey of children in the areas affected by the Great East Japan Earthquake, an increase in the prevalence of allergic diseases was observed. Disaster experience was associated with atopic dermatitis among boys, and with asthma among girls. Take measures to prevent indoor moisture and condensation when designing or moving into prefabricated temporary housing. Frequent airing out of futons (bedding), regular ventilation and cleaning of rooms, and the avoidance of moisture sources in the room can be useful when dealing with these issues.

Nation-wide health survey of nursery pupils

Subjects: 122,751 pupils

from 3,465 nurseries

Born in 2004: 53,747

Born in 2006: 69,004

Prevalence of allergic diseases

〈Atopic dermatitis〉

Among pupils unaffected by the disaster: 3.4%

Among pupils affected by the disaster: 4.8%

(1.4% increase)

〈Asthma〉

Among pupils unaffected by the disaster: 3.3%

Among pupils affected by the disaster: 6.3%

(3% increase)

*Statistically significant difference

There is a high risk of mold growing on the ceiling or under a futon/mattress in a prefabricated temporary housing unit (especially in bedrooms)

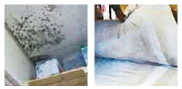

How to prevent mold and mite in prefabricated temporary housing units

Take extra care in the bedroom!

1 Inhibit dew formation and moisture in the house.

- Air the futon regularly.
- Do not use anything that may generate increased moisture.
- Ventilate rooms periodically.

2 Clean rooms frequently

- Clean rooms with a vacuum cleaner at least once a week.
- Wipe floors before vacuuming them.

3 Maintain a clean bedroom.

- Vacuum bedclothes at least once a week.
- Avoid placing stuffed toys or cushions around the bedroom.
- Do not use carpets and leave wooden flooring intact.

When cleaning rooms, be sure to keep the windows open for ventilation, and cover your mouth with a mask and towel.

※Reference

・Health Labour Sciences Research "Research on preventing asthma deaths and encouraging self-management in patients with bronchial asthma" Ken Ohta, National Hospital Organization Tokyo National Hospital, Naomi Tsurikisawa, National Hospital Organization Saitama National Hospital

・"Research on allergy risk assessment of fungi in emergency living environments in areas affected by the Great East Japan Earthquake and on prevention and hygiene control" Maiko Watanabe, National Institute of Health Sciences

POINT 2

Maintain good sleep.

Survey findings Sleep was negatively affected in the long term after the GEJE.

Recommendation Arrange for an optimal sleep environment and take time to relax before going to bed.

Sleep disturbance was observed among residents in the areas affected by the Great East Japan Earthquake even years after the event. Sleep maintains our mental and physical health. When sleep is disturbed, it can cause difficulty with concentration, and can lead to increased risk of mental health problems and increased vulnerability to illnesses. If you have difficulty with falling or maintaining sleep, try to create an optimal sleep environment (dark and quiet), and take time to relax before going to bed. One way to relax is to perform abdominal breathing exercises for a few minutes before falling asleep in bed. Focusing on your breath calms down your physical sensations, and helps you to fall asleep easier. Avoid having a nightcap (i.e., drinking alcohol) as it worsens the quality of sleep. In the morning, step out in the sun and activate your circadian rhythm. If you continue to have difficulty with sleep, seek professional help from health care providers of local government or clinics.

The sleep conditions of the affected residents after the Great East Japan Earthquake

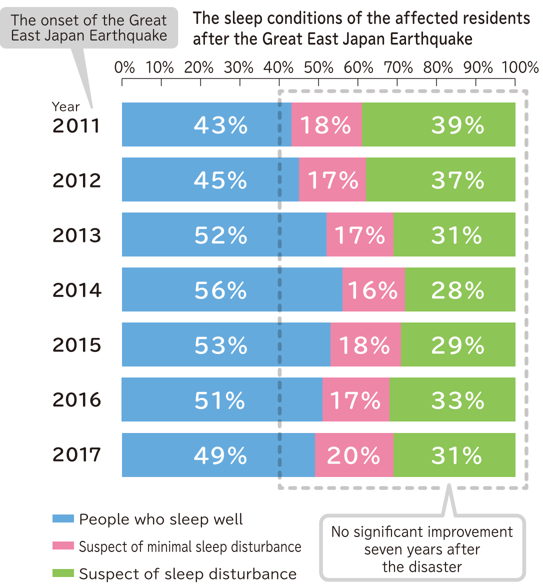

Try abdominal breathing!

1, 2, 3, (4), 5, 6, 7, 8, 9, 10

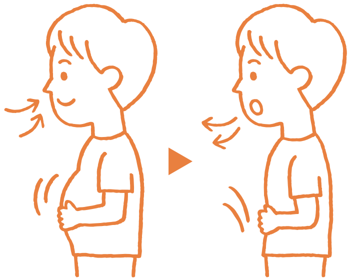

Breathe in slowly while counting 1, 2, and 3 and pushing out your stomach so that the abdomen expands when you have fully inhaled.
On count 4, stop breathing in.
Breathe out slowly while counting 5, 6, 7, 8, 9, and 10 and pulling in your stomach so that the stomach falls inward maximally when you have fully exhaled.

※Reference The 1st through 7th Surveys based on the joint project of Tohoku University and the Shichigahama town, Miyagi Prefecture "Shichigahama Health Promotion Project"

POINT 3

Maintain habitual physical activities.

Survey findings A higher percentage of overweight children was observed after the GEJE.

Recommendation Try to make a habit of walking.

The percentage of children found to be overweight significantly increased in the areas affected by the Great East Japan Earthquake compared to other areas. Adults who do not walk regularly also suffered more frequently from sleep disturbance, psychological distress, depressive states, and post-traumatic stress reactions. Unless you suffer from physical or mental exhaustion or are not feeling well, a habit of walking may improve your physical and mental health. Try to take time to go for a walk or run errands on foot and use stairs instead of the elevator. It is also useful to use a pedometer to become aware of your daily step counts.

The proportion of overweight nursery pupils (aged 3 and a half years to 4 and a half years) in three prefectures which were majorly damaged by the Great East Japan Earthquake (Iwate, Miyagi, and Fukushima) and the remaining prefectures.

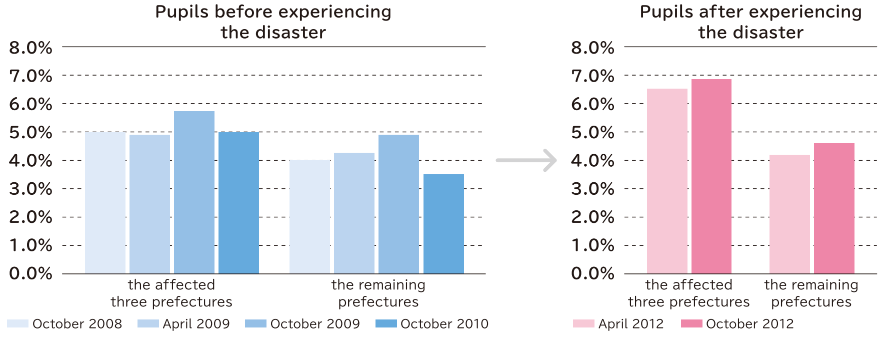

Significant increase in the proportion of overweight nursery pupils was observed in the three affected prefectures

※Reference
・Health Labour Sciences Research Project "Research on children's health in the areas affected by the Great East Japan Earthquake"
・The 1st through 7th Surveys based on the joint project of Tohoku University and the Shichigahama town, Miyagi Prefecture "Shichigahama Health Promotion Project"

POINT 4

Stay connected with others.

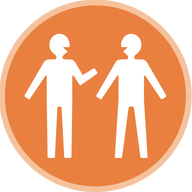

**Survey findings**	Staying connected with others helped to keep people mentally healthy after the GEJE.
**Recommendation**	Participate in gatherings and talk to others.

People who stay connected with others were found to be less prone to develop difficulty with sleep, psychological distress, depressive states, and post-traumatic stress reactions. Participating in gatherings and talking to neighbors is useful in keeping you and those around you emotionally healthy.

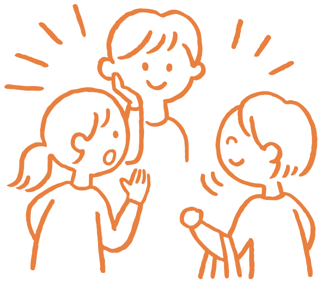

Social interactions and mental health conditions

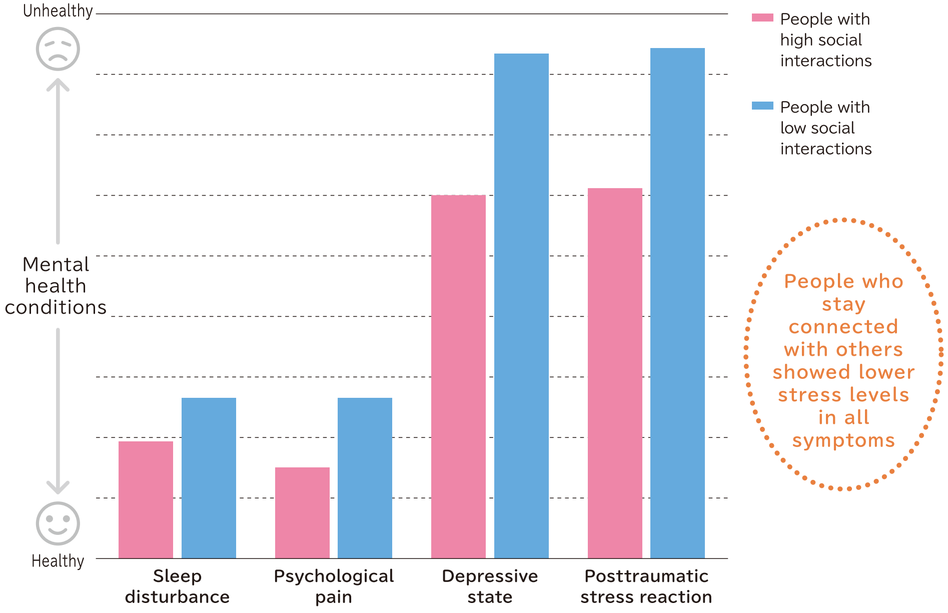

※Reference The 7th Surveys based on the joint project of Tohoku University and the Shichigahama town, Miyagi Prefecture "Shichigahama Health Promotion Project"

POINT 5

Avoid consuming too much alcohol.

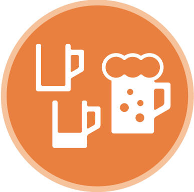

**Survey findings**	Heavy drinking contributed to emotional instability.
**Recommendation**	Cut down on alcohol to a moderate amount.

A moderate amount of alcohol is no more than 20 g of pure alcohol a day on average. This is equivalent to a medium-sized bottle or a 500-ml can of beer with an alcohol content of 5%, a 350-ml can of 7% "chuhai (shōchū highball)", a little less than 1 "go" (180 ml) of 15% sake, or a glass and a half (200 ml) of 12% wine. A daily intake that exceeds three times this amount is referred to as heavy drinking, and temperance is recommended for healthier living. The disaster-affected residents who drank heavily were found to be more prone to develop difficulty in sleep, psychological distress, depressive states, and post-traumatic stress reactions. Heavy drinking has adverse effects on your sleep and mental health. If you are a heavy drinker, seek professional help to cut down the amount of your alcohol intake.

The amount of alcohol intake per day and mental health conditions

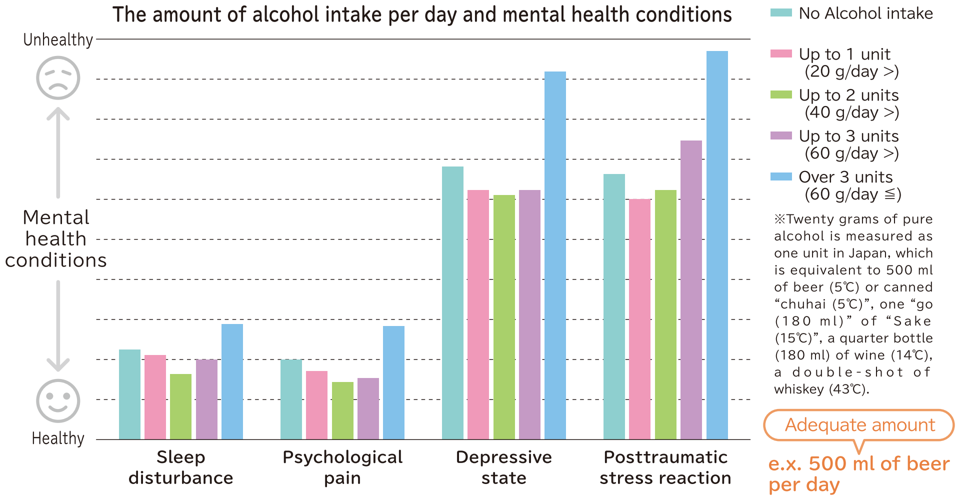

※Reference The 7th Surveys based on the joint project of Tohoku University and the Shichigahama town, Miyagi Prefecture "Shichigahama Health Promotion Project"

POINT 6

Take advantage of health checkups and cancer screenings.

**Survey findings**	The rate of gynecological exams decreased after the GEJE among young adults.
**Recommendation**	Once things settle down after a disaster, make sure to have checkups.

After a disaster, you may become too busy putting your life back together to properly take care of your own health. In a survey conducted in the areas affected by the Great East Japan Earthquake, the rate of gynecological exams was found to be lower than the standards before the earthquake among young adults. Once things have settled down in the aftermath of a disaster, be sure to have regular checkups.

Screening rate **Screening rates of uterine cancer among young women** (20 y.o. to 49 y.o.)

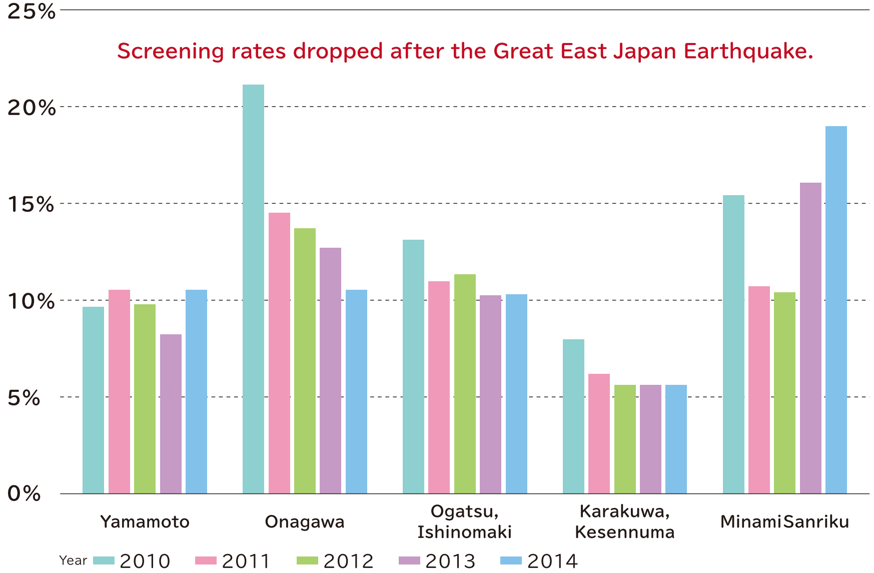

(Adapted from the Miyagi Cancer Society Annual Report)

POINT 7

Prevent infections.

**Previous findings**	Infections spread quickly in shelters and temporary housing.
**Recommendation**	Follow the "Eight rules to prevent infectious diseases."

In shelters and temporary housing, infections spread quickly through the delivery of food supplies and the use of soup kitchens and communal bathrooms, as well as due to limited living spaces. Standard measures are effective in preventing infections even in the extraordinary environments after a disaster. Follow the "Eight rules to prevent infectious diseases."

■ Eight rules to prevent infectious diseases (TOHOKU INFECTIONS DISEASE CRISIS CONTROL NETWORK, MAY, 2015)

What to do to prevent infection (to the extent possible at your location)

① If possible, make sure your meal is thoroughly cooked.
② Drink only the water designated for drinking (drinkable water) and use a clean glass/cup.
③ Wash your hands before your meal, and after going to the restroom.
(Wash with water or use disinfectant alcohol for hands.)
④ Use only the designated area to discard diapers and then wash your hands.

What to do if you have symptoms

⑤ Cover your mouth with your hand when you cough or sneeze.
(put on a mask - - if available.)
⑥ If you have a fever, sore throat, cough, injury or diarrhea, or if you are vomiting, and especially if others around you have similar symptoms, consult the doctor, nurse or authority.
⑦ If you have a fever and/or cough or if you are looking after anyone with such symptoms, wear a mask as much as possible.
⑧ If you or someone has any of the following symptoms, appropriate treatment may need to be sought at a medical facility. Please consult the doctor, nurse, or authority.

- heavy coughing or coughing up yellow sputum.
- having difficulty breathing or heavy breathing.
- feeling very weak/exhausted or looking very pale.

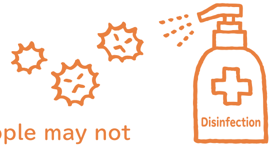

Caution Symptoms among children and elderly people may not appear right away- pay attention and please report if you notice anything unusual health-wise among them.

POINT 8

Be cautious of the impact of a disaster on your mental health.

Previous findings Stress reactions to a disaster persisted for a long time after the GEJE.

Recommendation Find someone to share your problems with.

The Great East Japan Earthquake affected both mental and physical health. Mental conditions such as depressive states and post-traumatic stress reactions persisted among the affected communities for a long time after the disaster. When you are suffering from painful flashbacks of your experiences with the disaster, feeling nervous, and/or avoiding anything that reminds you of the disaster, please do not put pressure on yourself. Please rest appropriately and find someone to talk to. Instead of keeping it to yourself, share your pains and problems with those whom you feel safe.

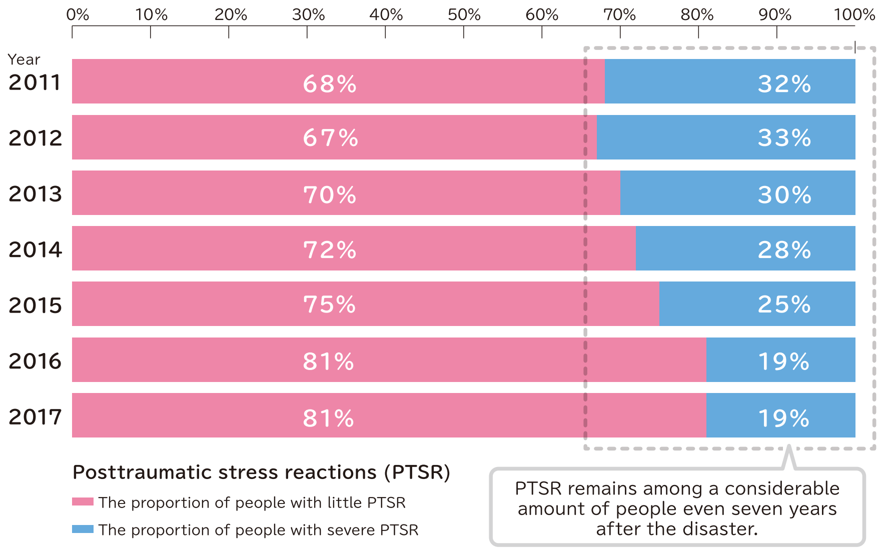

※Reference The 1st through 7th Surveys based on the joint project of Tohoku University and the Shichigahama town, Miyagi Prefecture "Shichigahama Health Promotion Project"

POINT 9

Be mindful of expectant and breastfeeding mothers.

Previous findings Maternity passbooks were lost in the tsunami during the GEJE.

Recommendation Carry your maternity passbook with you at all times, and keep the contact information of your hospital/clinic.

During the Great East Japan Earthquake, some expectant mothers lost their maternity passbooks in the tsunami. Expectant mothers are advised to carry their maternity passbooks at all times in preparation of a disaster. Also, be sure to keep the contact information of your hospital/clinic handy. Even if you lose your passbook, your record may be held at your hospital/clinic.

To expectant mothers

When you arrive at a shelter, let the person in charge and people around you know that you are expecting a baby. Also, those providing support at shelters should always check if there are any expectant mothers present. Even if expectant mothers appear healthy, abdominal pain, bleeding, or other changes in health conditions can suddenly occur, which can threaten the lives of both the mother and the fetus. Please be sure to direct particular attention to expectant mothers.

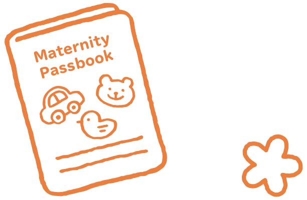

To mothers with small children

Mothers who breastfeed their children are recommended to continue with breastfeeding even when at a shelter. When preparing baby formula, you need to use safe water and to sterilize (disinfection by boiling) the bottle/bottle nipple. If, by any chance, these are not available, use a clean paper cup to prevent infections.

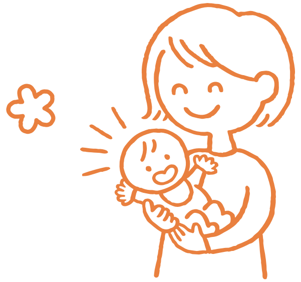

Supplement: Supplementary file 1 — Supplementary Information. [file 41598_2024_52121_MOESM1_ESM.pdf]
